# Chimeric virus-like particles presenting common neutralizing epitopes of enterovirus 71

**Published:** 2011-01-10

**Authors:** Bo Wang, Qingwei Liu, Jinping Shi, Yicun Cai, Zhong Huang

**Affiliations:** 1 Key laboratory of Molecular Virology & Immunology, Institut Pasteur of Shanghai, Chinese Academy of Sciences, Shanghai 200025, PR China

## 

To develop an effective vaccine against multiple genotypes of enterovirus 71 (EV71), we generated chimeric virus-like particles (VLPs) repetitively displaying the common neutralizing epitopes of EV71 and evaluated their immunogenicities in mice. The two conserved epitopes, encompassing amino acids 163-177 and 208-222 of VP1 of EV71, were fused to hepatitis B core antigen (HBcAg) and expressed in *E.coli*. The resulting fusion proteins were found to assemble into chimeric VLPs. Both unmodified HBcAg and chimeric VLPs induced HBcAg-specific antibody responses in mice, however, only chimeric VLP-immunized sera possessed EV71 epitope-specific IgG antibodies and efficiently neutralized different EV71 strains. Collectively, our results indicate that the chimeric VLP is capable of eliciting broadly neutralizing antibody responses and is therefore a promising EV71 vaccine candidate.

